# Parental Support Is Associated with Moderate to Vigorous Physical Activity among Chinese Adolescents through the Availability of Physical Activity Resources in the Home Environment and Autonomous Motivation

**DOI:** 10.3390/children9091309

**Published:** 2022-08-28

**Authors:** Jing Zeng, Nan Qiu, Brianna N. Leitzelar, Jialin Fu, Yechuang Wang, Fang Liang, Kai Ding, Justin B. Moore, Yuliang Zou, Rui Li

**Affiliations:** 1 Department of Healthcare Management, School of Public Health, Wuhan University, Wuhan 430071, China; 2 Department of Social Sciences and Health Policy, Division of Public Health Sciences, Wake Forest University School of Medicine, Winston-Salem, NC 27101, USA; 3 Department of Implementation Science, Division of Public Health Sciences, Wake Forest University School of Medicine, Winston-Salem, NC 27101, USA; 4 School of Nursing, Wuhan University, Wuhan 430071, China

**Keywords:** moderate to vigorous physical activity, parental support, home physical activity environment, autonomous motivation, adolescent

## Abstract

This study aimed to use a structural equation model (SEM) to determine the association between parental support and moderate to vigorous physical activity (MVPA) among Chinese adolescents and whether the availability of physical activity (PA) resources in the home environment and autonomous motivation of adolescents mediated the association. Data were collected using questionnaires extracted from the Family Life, Activity, Sun, Health, and Eating (FLASHE) study. A final analytical sample of 3738 adolescents was enrolled. A SEM was performed to evaluate the hypothesized associations. It was found that parental support was not only positively directly but also indirectly associated with MVPA in Chinese boys through the home environment (i.e., availability of PA resources) and the autonomous motivation of adolescents. It is worth noting that the above relationships also exist in Chinese girls, except for the regulatory role of autonomous motivation. These findings suggest that future interventions for increasing adolescents’ MVPA should focus on health education for parents to provide more PA resources in the home environment and adequately mobilize children’s autonomous motivation.

## 1. Introduction

Regular moderate to vigorous physical activity (MVPA) is essential for the health and well-being of adolescents and promotes positive health leading into adulthood [1]. Youths who engage in regular MVPA are more likely to display favorable body composition, cardiorespiratory and musculoskeletal fitness, academic achievement, and cognitive abilities [2,3]. Conversely, insufficient physical activity (PA) is linked to a lower quality of life and the development of chronic diseases, such as ischemic heart disease, diabetes and breast cancer in adulthood [4,5]. A minimum of 60 min of MVPA per day is urged by the World Health Organization for adolescents [6]. However, less than one-third of Chinese adolescents meet this recommendation [7]. Therefore, it is important to identify the modifiable correlates of MVPA in Chinese adolescents in order to develop targets for future intervention.

Parents are instrumental in shaping their children’s health behaviors and can influence their children directly through their actions (i.e., modeling PA) [8,9]. In the context of adolescent MVPA, parental support is one potentially modifiable correlated factor [10]. Parental support (i.e., the tangible and intangible mechanisms that facilitate their child’s MVPA) has been associated with adolescent MVPA [11]. For example, encouragement from parents was positively related to participation in MVPA in Canadian and Australian adolescents [10,11,12]. At the same time, parents are principally responsible for structuring the home environment [13]. The home environment can positively affect children’s social interactions and promote healthy behaviors, such as MVPA [14,15]. A five-year cohort study of fifth graders in South Carolina, USA, demonstrated that adequate PA equipment at home was positively correlated with an adolescent’s daily PA [16]. Parental support and the home environment (i.e., availability of resources in the home environment) may be key predictors of adolescent PA behavior.

Autonomous motivation also plays a role in adolescent PA behavior [17]. The self-determination theory (SDT) of behavior change defines motivation as the intention of an individual to perform an action and is a predictor of human behaviors [18]. Autonomous motivation means that people are fully aware of the value of a behavior and incorporate it into their self-consciousness [19]. A prospective study showed that children’s motivation, based on changes in SDT constructs, led to changes in the behaviors associated with PA, and children who maintained high autonomous motivation also had a high level of PA [20]. Several studies have reported that autonomous motivation strongly correlates positively with MVPA, and a good autonomous motivation can predict a higher level of MVPA in adolescents [17,21,22].

At the same time, parental support and the home environment may influence adolescents’ autonomous motivation. For example, previous research demonstrated that autonomous support from a significant other might affect one’s autonomous motivation [23]. Further, the home environment was associated with motivation, which in turn influenced the PA levels in a sample of adults from the USA [24]. However, no association was found between teenagers’ autonomous motivation and the home environment (i.e., the availability of PA resources) in a sample of Belgian teenagers [25]. Further study is warranted to understand these relationships in adolescents.

Furthermore, most prior studies have concentrated on Western developed countries (i.e., the USA or Europe). Since Chinese adolescents are under more academic pressure compared with their Western peers [26], their time to participate in PA is restricted, so their responses to PA-related factors may differ. Further, a study has confirmed that the PA levels of Chinese children are significantly lower than that of children in western high-income countries [27]; thus, there is a need to investigate the issue surrounding the influencing factors related to the PA of Chinese adolescents.

Understanding the relationships between parental support, the home environment (i.e., the availability of PA resources), autonomous motivation, and MVPA will provide important insight into adolescent PA behavior. Most of the previous studies have examined the independent association between each construct and MVPA, but few studies targeting Chinese populations have jointly explored the mediating role of the above factors. Therefore, structural equation modeling (SEM) can be used to test the direct and indirect effects of variables. This study examined the concurrent relationships between parental support, the home environment (i.e., availability of PA resources), autonomous motivation, and MVPA in Chinese adolescents. Specifically, due to the consistent differences in PA behavior between boys and girls [28], we examined these variables in each gender using SEM. We hypothesized that support from parents correlated directly with adolescents’ MVPA and was indirectly related to adolescent MVPA through the home environment (i.e., the availability of PA resources) and autonomous motivation. 

## 2. Methods

### 2.1. Participants

In October 2019, 4519 students were invited to participate in a cross-sectional study from a secondary school in Wuhan, China. The study was carried out in light of the Declaration of Helsinki, and a Wuhan University Ethics Board grant of ethical approval was obtained (ethical approval code: 2019YF2056). With the consent of the school and parents, informed consent was signed by all youth before their participation. Paper questionnaires were distributed at the school on a class-by-class basis, which participants could complete within 15 min. The investigators introduced the study’s purpose, content and confidentiality commitment to the students and withdrew it after the participants had completed the questionnaire. In total, 4027 students aged 10–19 years were eligible and consented to complete this study. The participants who had missing data on MVPA (n = 157) and parental support (n = 132) were excluded (n = 289, 7.18%). Thus, the analytical samples were taken from 3738 subjects.

### 2.2. Measures

#### 2.2.1. Reliability and Validity of the Questionnaire

MVPA, parental support, the home environment (i.e., the availability of PA resources), and autonomous motivation were assessed using questionnaires derived from the FLASHE study [29]. Using the questionnaires developed by the United States National Cancer Institute, the FLASHE study assessed cancer-related behaviors (e.g., PA) in parent-child dyads, and further detail can be found elsewhere [29]. The questionnaires used in the current study were translated to Chinese and have been shown to be reliable and valid [30]. Further, statistics from the current study suggest the measures are reliable (Cronbach’s alpha MVPA = 0.78, Cronbach’s alpha parental support = 0.89, Cronbach’s alpha autonomous motivation = 0.77, Cronbach’s alpha availability of PA resources in the home environment = 0.83, Kaiser–Meyer–Olkin = 0.87, *p* Bartlett < 0.001).

#### 2.2.2. MVPA

MVPA was assessed with the Youth Activity Profile (YAP), which assesses the total time spent in MVPA (during school, outside of school and at the weekend) over the past week [31]. Multiplying the predicted percentage time in MVPA from YAP by the participants’ self-reported respective section time (in minutes) gives the predicted weekly minutes of activity [29]. In the current study, MVPA is regarded as a continuous variable based on the above calculation.

#### 2.2.3. Parental Support

We measured parental support to assess the degree to which the participants experience parental support for engaging in PA, using six items: “a. My parent(s) have to make sure that I get enough physical activity”; “b. My parent(s) take me places where I can be physically active ”; “c. My parent(s) and I decide together how much physical activity I have to do”; “d. My parent(s) make me exercise or go out and play”; “e. My parent(s) try to be physically active when I’m around”; “f. It’s okay for my parent(s) to make rules about how much time I spend being physically active/playing”. Answers to these items were indicated on a 5-point Likert scale (1 “strongly disagree” to 5 “strongly agree”).

#### 2.2.4. Autonomous Motivation

Autonomous motivation was assessed by asking two questions: “I have thought about it and decided that I want to exercise”, and “It is an important thing for me to do”. The answers to these items were indicated on a 5-point Likert scale (1 “strongly disagree” to 5 “strongly agree”). 

#### 2.2.5. Availability of Physical Activity Resources in the Home Environment

Questions assessing the home environment (i.e., the availability of PA resources) included the availability of eight types of PA equipment: “a. Bicycle. Don’t count stationary bikes”; “b. Basketball hoop”; “c. Sports equipment like balls, racquets, bats and sticks”; “d. Skateboard or scooter”; “e. Weight lifting equipment”; “f. Cardio equipment like treadmills, stationary bicycles, step climbers, elliptical machines, rowing machines, etc.”; “g. Active gaming like Wii or Xbox Kinect”; “h. Exercise videos or DVDs”. The answer options were the respective frequency of use: not available = 1, available but never used = 2, use once a month or less = 3, use once every other week = 4, use once a week or more = 5.

### 2.3. Analysis

Statistical analyses were conducted using SPSS 26.0 (IBM, Armonk, NY, USA) and the MPLUS software 8.3. Multiple imputations were used to complete the missing data of the participant’s basic information (i.e., 23 data were missing for the gender variables, 12 data were missing for the children’s educational level variables, 152 data were missing for the residence variables, 409 data were missing for the BMI z-score variables, 76 data were missing for the father’s educational level variables, 71 data were missing for the mother’s educational level variables, 279 data were missing for the household monthly income variables and 9 data were missing for the availability of PA resources in the home environment variables) that included 3738 participants since the missing data were considered missing at random [29]. The descriptive statistics were calculated for participants’ demographic characteristics, time spent in MVPA, parental support, the home environment (i.e., the availability of PA resources), and autonomous motivation. The continuous variables with non-normal distributions were represented as the median and interquartile ranges, and the categorical variables were represented as numbers and proportions. The gender-based differences were tested using the Kruskal–Wallis (KW) test. Spearman correlation analyses were performed to quantify the correlations among variables.

**Figure 1:**
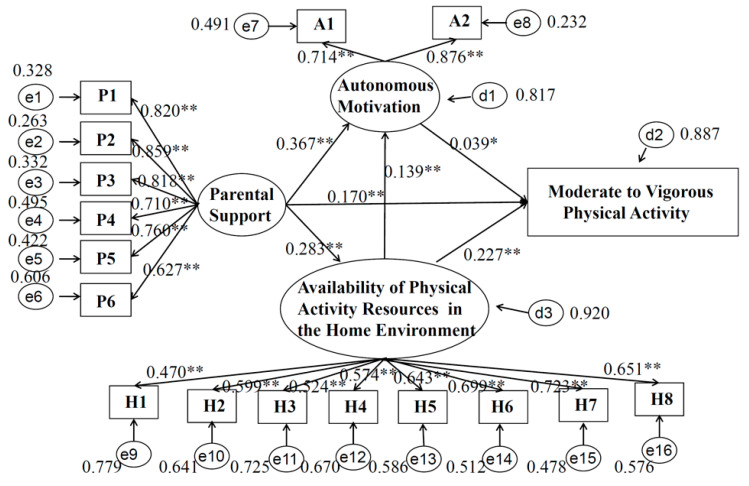
Model of moderate to vigorous physical activity in the total adolescent sample. Note: ** *p* < 0.001, * *p* < 0.05. P1, “a. My parent(s) have to make sure that I get enough physical activity”; P2, “b. My parent(s) take me places where I can be physically active ”; P3, “c. My parent(s) and I decide together how much physical activity I have to do”; P4, “d. My parent(s) make me exercise or go out and play”; P5, “e. My parent(s) try to be physically active when I’m around”; P6, “f. It’s okay for my parent(s) to make rules about how much time I spend being physically active/playing”; A1, “I have thought about it and decided that I want to exercise”; A2, “It is an important thing for me to do”; H1, “a. Bicycle. Don’t count stationary bikes”; H2, “b. Basketball hoop”; H3, “c. Sports equipment like balls, racquets, bats and sticks”; H4, “d. Skateboard or scooter”; H5, “e. Weight lifting equipment”; H6, “f. Cardio equipment like treadmills, stationary bicycles, step climbers, elliptical machines, rowing machines, etc.”; H7, “g. Active gaming like Wii or Xbox Kinect”; H8, “h. Exercise videos or DVDs”.

The sample size required at least 500 participants in the SEM and a 10:1 or 20:1 ratio between the number of subjects and the free parameters to be estimated in the model [32]. The current study included 3738 subjects, and the number of free parameters to be estimated was 56. In this way, the sample size is sufficient. The normality of all data in SEM was tested by skewness and kurtosis in the MPLUS software, and the criteria for normality were the variables with skewness between +3 and −3 and kurtosis between +10 and −10, the variables met this standard in this model. The maximum likelihood method was used to evaluate the SEM. As mentioned in the introduction, the SEM was used to test the hypotheses of parental support and adolescent MVPA and to examine the regulatory role of variables, such as autonomous motivation and the home environment (i.e., the availability of PA resources). The measurement and structural models in the SEM are described in detail in Figure 1. The analyses were conducted in the total sample and separately by gender.

For the evaluation of the SEM based on the fitting quality criteria, the ideal quality adjustment parameters were as follows: the comparative fit index (CFI) and a Tucker Lewis index (TLI) greater than 0.9 indicated a good fit, and the closer to 1, the better the fitting. A root mean square error of approximation (RMSEA) of less than 0.01, 0.05 and 0.08, respectively, indicate that the fit is good, the fitting is excellent and acceptable, and finally, a standardized root mean square residual (SRMR) of less than 0.8 is regarded as a good fit [33]. The statistical significance threshold was set at *p* < 0.05, using the double-tailed test and 95% confidence intervals (CI) excluding 0.

## 3. Results

The bivariate correlation between parental support, the home environment (i.e., the availability of PA resources), autonomous motivation and MVPA in the model is shown in Table 1. The correlation between MVPA and all items was statistically significant.

Figure 1 shows the standardized coefficients from the model. Parental support was positively correlated with the home environment (i.e., availability of PA resources) (b = 0.283, 95% CI = 0.249, 0.317), autonomous motivation (b = 0.367, 95% CI = 0.331, 0.403), and MVPA (b = 0.170, 95% CI = 0.134, 0.207). The home environment (i.e., the availability of PA resources) was positively associated with autonomous motivation (b = 0.139, 95% CI = 0.101, 0.177) and MVPA (b = 0.227, 95% CI = 0.192, 0.261). Autonomous motivation was positively associated with MVPA (b = 0.039, 95% CI = 0.001, 0.077). In the model of total population (3738), four fitting indicators have been identified as follows: CFI = 0.925, TLI = 0.911, SRMR = 0.048, RMSEA = 0.066 (95% CI = 0.063, 0.068). The above indicators show that the model fits well. In addition, six endogenous variables that were supported by parents, eight endogenous variables of the home environment (i.e., the availability of PA resources) and two endogenous variables of autonomous motivation all contributed significantly to the latent variables of parental support, the home environment (i.e., availability of PA resources) and autonomous motivation, respectively.

**Figure 2:**
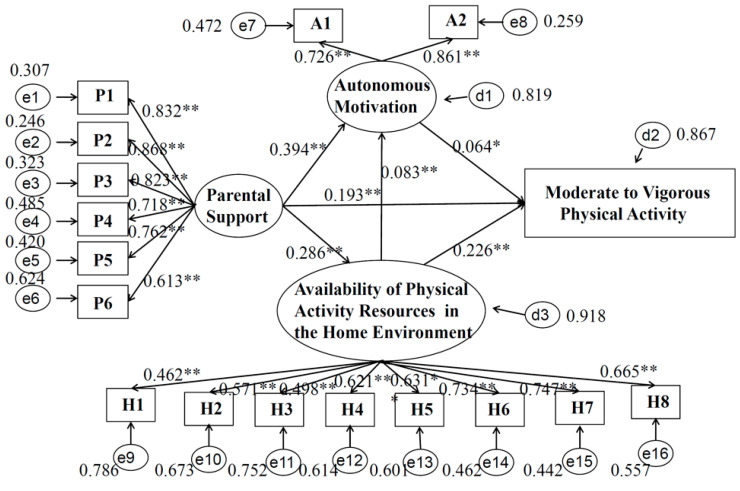
Model of moderate to vigorous physical activity for boys. Note: Note: ** *p* < 0.001, * *p* < 0.05. P1, “a. My parent(s) have to make sure that I get enough physical activity”; P2, “b. My parent(s) take me places where I can be physically active ”; P3, “c. My parent(s) and I decide together how much physical activity I have to do”; P4, “d. My parent(s) make me exercise or go out and play”; P5, “e. My parent(s) try to be physically active when I’m around”; P6, “f. It’s okay for my parent(s) to make rules about how much time I spend being physically active/playing”; A1, “I have thought about it and decided that I want to exercise”; A2, “It is an important thing for me to do”; H1, “a. Bicycle. Don’t count stationary bikes”; H2, “b. Basketball hoop”; H3, “c. Sports equipment like balls, racquets, bats and sticks”; H4, “d. Skateboard or scooter”; H5, “e. Weight lifting equipment”; H6, “f. Cardio equipment like treadmills, stationary bicycles, step climbers, elliptical machines, rowing machines, etc.”; H7, “g. Active gaming like Wii or Xbox Kinect”; H8, “h. Exercise videos or DVDs”.

**Figure 3:**
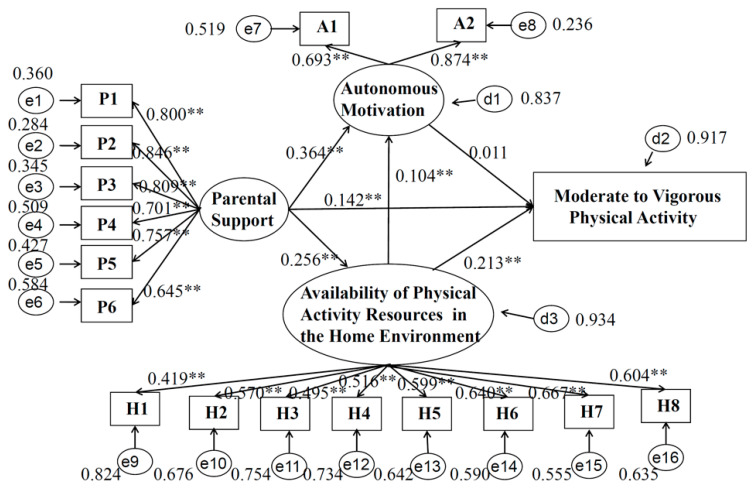
Path model of moderate to vigorous physical activity for girls. Note: ** *p* < 0.001. P1, “a. My parent(s) have to make sure that I get enough physical activity”; P2, “b. My parent(s) take me places where I can be physically active ”; P3, “c. My parent(s) and I decide together how much physical activity I have to do”; P4, “d. My parent(s) make me exercise or go out and play”; P5, “e. My parent(s) try to be physically active when I’m around”; P6, “f. It’s okay for my parent(s) to make rules about how much time I spend being physically active/playing”; A1, “I have thought about it and decided that I want to exercise”; A2, “It is an important thing for me to do”; H1, “a. Bicycle. Don’t count stationary bikes”; H2, “b. Basketball hoop”; H3, “c. Sports equipment like balls, racquets, bats and sticks”; H4, “d. Skateboard or scooter”; H5, “e. Weight lifting equipment”; H6, “f. Cardio equipment like treadmills, stationary bicycles, step climbers, elliptical machines, rowing machines, etc.”; H7, “g. Active gaming like Wii or Xbox Kinect”; H8, “h. Exercise videos or DVDs”.

The models of standardized coefficients by gender are presented in Figure 2 (boys) and Figure 3 (girls), respectively. For all students (regardless of gender), parents’ support was positively correlated with the home environment (i.e., the availability of PA resources) (b _boy_ = 0.286, 95% CI _boy_ = 0.239, 0.332; b _girl_ = 0.256, 95% CI _girl_ = 0.204, 0.309), and with autonomous motivation (b _boy_ = 0.394, 95% CI _boy_ = 0.344, 0.443; b _girl_ = 0.364, 95% CI _girl_ = 0.311, 0.417). The home environment (i.e., the availability of PA resources) was correlated with autonomous motivation (b _boy_ = 0.083, 95% CI _boy_ = 0.030, 0.135; b _girl_ = 0.104, 95% CI _girl_ = 0.047, 0.161) and with MVPA (b _boy_ = 0.226, 95% CI _boy_ = 0.179, 0.272; b _girl_ = 0.213, 95% CI _girl_ = 0.160, 0.265). For boys, autonomous motivation was positively correlated with MVPA (b _boy_ = 0.064, 95% CI _boy_ = 0.012, 0.117), while girls’ autonomous motivation showed no statistically significant relation with MVPA (b _girl_ = 0.011, 95% CI _girl_ = −0.046, 0.068). Specifically, although parental support was directly associated with MVPA in males and females, the effect was different (b _boy_ = 0.193, 95% CI _boy_ = 0.143, 0.243; b _girl_ = 0.142, 95% CI _girl_ = 0.087, 0.196).

In the gender-specific SEM, the fitting results of the two models are as follows: CFI _boy_ = 0.915, TLI _boy_ = 0.899, SRMR _boy_ = 0.052, RMSEA _boy_ = 0.072 (95% CI = 0.068, 0.075). CFI _girl_ = 0.932, TLI _girl_ = 0.919, SRMR _girl_ = 0.046, RMSEA _girl_ = 0.058 (95% CI = 0.055, 0.062). The above indicators show that the two models fit well.

## 4. Discussion

This research tested a novel hypothesis regarding the direct action of parental support on adolescent MVPA and the mediating effects of the home environment (i.e., the availability of PA resources) and autonomous motivation in a large sample of Chinese adolescents. Our results revealed a direct effect of parental support on adolescent MVPA and indirect effects of parental support through the home environment (i.e., the availability of PA resources) and autonomous motivation, and these relationships differed by gender.

Parental support was directly correlated with adolescents’ MVPA, which is consistent with the results of other cross-sectional studies [34,35]. These findings imply that participation in MVPA by adolescents is heavily dependent on the support of their parents. Additionally, boys reported significantly more parental support than girls. This observed difference is consistent with a cross-sectional study from the UK [36]. Previous research demonstrated that the influence of parental role models was stronger among same-sex parents and children, such that fathers mainly influenced their sons while mothers mainly influenced their daughters [37,38]. In China, most mothers play the role of family maintainer, which reduces their time to participate in PA and thereby weakens their ability to provide PA support and influence their child’s PA [39]. Perhaps this explains our finding that girls reported less support than boys.

The home environment (i.e., the availability of PA resources) moderates the relationship between parental support and adolescent’ MVPA. This suggests that the more PA support provided by parents, for example, the greater the availability of PA resources at home, the more adolescents participated in MVPA. This relationship is consistent with a longitudinal study among fifth-grade children in South Carolina [16]. What is more, the current results demonstrate that boys reported more PA resources in the home environment than girls. Since PA resources configured in the home may be more preferred by boys, and girls’ PA behaviors are associated with the amount and variety of exercise equipment [40], the lack of girl-preferred exercise equipment in the home (e.g., jump ropes and yoga mats) makes boys report greater equipment resources in the home than girls. Increasing the accessibility of PA resources may have a differential impact on MVPA participation for boys and girls between the ages of 12 and 18 [41].

One indirect path demonstrated that autonomous motivation mediated the relationship between parental support and adolescents’ MVPA in boys rather than girls. This suggests that higher levels of parental support are related to higher autonomous motivation, which then influences PA behavior. Moreover, both parental support and adolescents’ autonomous motivation have been shown to have a lasting impact on adolescents’ MVPA [18,42]. However, in the current sample of girls, there is no statistical correlation between autonomous motivation and MVPA. The reasons may be various. Based on SDT theory, internalizing the value of MVPA outcomes by emphasizing the importance of MVPA to health, physical function and quality of life for individuals can not only improve MVPA-related well-being but also have greater persistence [43]. In the traditional concept, PA tend to be masculine or, in principle, PA will be arranged for males [44], which greatly weakens the internalized value of PA for girls. Therefore, it may be crucial to promote adolescent MVPA by encouraging parents to provide more support, such as providing more diversified opportunities for PA, offering positive feedback and timely encouragement, which might increase children’s autonomous motivation, especially for girls.

The home environment (i.e., the availability of PA resources) was also correlated with autonomous motivation, suggesting that the home environment may influence autonomous motivation for MVPA. A previous study demonstrated that high school girls did not engage in out-of-school MVPA in the neighborhood environment [45]. Thus, the home environment might be especially poignant for girls. This relationship emphasized that the more PA resources adolescents obtained in the home environment, the stronger their intention to be active (i.e., autonomous motivation), and then the higher level of MVPA they participated in.

The findings were discussed from a theoretical and practical perspective, which has enlightening significance for intervention development. In order to improve the MVPA level of teenagers, health education should be given to parents so that they can set a good example of PA for their children. In addition to providing logistical support (e.g., transporting children to sports venues) and sharing PA with their children, parents can also provide PA facilities in the home (e.g., jump ropes, yoga mats, treadmills and ball equipment). Similarly, parents should fully mobilize their children’s autonomous motivation and praise and encourage their children’s sports behavior.

Although prior studies have examined the impact of parental support on MVPA, the present study evaluated the home environment (i.e., the availability of PA resources) and autonomous motivation as mediating factors using path analysis. Several limitations should be acknowledged. First, the samples were comparatively large, but they came from a single school, limiting the generalizability of the results to other adolescents. Second, MVPA was measured using self-report data; compared with objective measurement, self-report data is subjective and may introduce bias [46,47]. However, because the self-administered questionnaire is relatively inexpensive and acceptable, it may be suitable for studies with large sample sizes [47]. Third, there were gender differences in the types of sports equipment representing the home environment (i.e., the availability of PA resources) in the questionnaire, and boys prefer these sports equipment, which may bias the results. Lastly, the cross-sectional nature of this study hinders the derivation of causality.

## 5. Conclusions

The current study examined the association between parental support and MVPA as well as potential mediating factors (i.e., the availability of PA resources in the home environment and autonomous motivation) among Chinese adolescents. Parental support was not only directly but indirectly positively associated with MVPA among Chinese adolescents. The indirect associations demonstrated that the home environment (i.e., the availability of PA resources) and autonomous motivation mediated the relationship between parental support and adolescent MVPA for boys, and the home environment (i.e., the availability of PA resources) mediated the relationship between parental support and adolescent MVPA for girls. Future MVPA interventions targeted at increasing adolescent MVPA should focus on enhancing parental support through offering health education to provide more PA resources in the home environment and to support their children’s autonomous motivation.

## Figures and Tables

**Table 1:** Correlation between the factors of parental support, availability of physical activity resources in the home environment and autonomous motivation and moderate to vigorous physical activity.

	A1	A2	H1	H2	H3	H4	H5	H6	H7	H8	P1	P2	P3	P4	P5	P6	MVPA
A1	1																
A2	0.61 **	1															
H1	0.13 **	0.14 **	1														
H2	0.11 **	0.16 **	0.33 **	1													
H3	0.23 **	0.26 **	0.37 **	0.38 **	1												
H4	0.03	0.06 **	0.32 **	0.32 **	0.32 **	1											
H5	0.08 **	0.13 **	0.25 **	0.40 **	0.28 **	0.36 **	1										
H6	0.03 *	0.08 **	0.25 **	0.38 **	0.27 **	0.35 **	0.46 **	1									
H7	−0.02	0.06 **	0.26 **	0.42 **	0.24 **	0.38 **	0.45 **	0.57 **	1								
H8	0.04 *	0.09 **	0.21 **	0.34 **	0.27 **	0.34 **	0.40 **	0.44 **	0.53 **	1							
P1	0.29 **	0.34 **	0.17 **	0.15 **	0.26 **	0.11 **	0.10 **	0.10 **	0.08 **	0.11 **	1						
P2	0.28 **	0.30 **	0.16 **	0.14 **	0.25 **	0.11 **	0.07 **	0.10 **	0.07 **	0.11 **	0.70 **	1					
P3	0.20 **	0.24 **	0.14 **	0.12 **	0.20 **	0.11 **	0.08 **	0.14 **	0.12 **	0.13 **	0.62 **	0.66 **	1				
P4	0.29 **	0.30 **	0.14 **	0.10 **	0.22 **	0.10 **	0.05 **	0.05 **	0.03	0.07 **	0.57 **	0.64 **	0.50 **	1			
P5	0.19 **	0.23 **	0.12 **	0.14 **	0.20 **	0.11 **	0.09 **	0.12 **	0.13 **	0.13 **	0.56 **	0.60 **	0.61 **	0.50 **	1		
P6	0.12 **	0.17 **	0.14 **	0.11 **	0.15 **	0.10 **	0.08 **	0.11 **	0.12 **	0.14 **	0.45 **	0.42 **	0.59 **	0.30 **	0.51 **	1	
MVPA	0.10 **	0.15 **	0.21 **	0.14 **	0.22 **	0.21 **	0.09 **	0.14 **	0.15 **	0.13 **	0.20 **	0.20 **	0.22 **	0.14 **	0.17 **	0.22 **	1

Note: ** *p* < 0.001, * *p* < 0.05. MVPA, moderate to vigorous physical activity. P1, “a. My parent(s) have to make sure that I get enough physical activity”; P2, “b. My parent(s) take me places where I can be physically active ”; P3, “c. My parent(s) and I decide together how much physical activity I have to do”; P4, “d. My parent(s) make me exercise or go out and play”; P5, “e. My parent(s) try to be physically active when I’m around”; P6, “f. It’s okay for my parent(s) to make rules about how much time I spend being physically active/playing”; A1, “I have thought about it and decided that I want to exercise”; A2, “It is an important thing for me to do”; H1, “a. Bicycle. Don’t count stationary bikes”; H2, “b. Basketball hoop”; H3, “c. Sports equipment like balls, racquets, bats and sticks”; H4, “d. Skateboard or scooter”; H5, “e. Weight lifting equipment”; H6, “f. Cardio equipment like treadmills, stationary bicycles, step climbers, elliptical machines, rowing machines, etc.”; H7, “g. Active gaming like Wii or Xbox Kinect”; H8, “h. Exercise videos or DVDs”. There were significant gender discrepancies in the primary variables of interest, which are shown in Table 2. Boys reported more time for participating in MVPA than girls [812.49 (686.94, 945.91) min/week vs. 787.34 (686.94, 945.91) min/week, *p* < 0.01] and reported higher scores in parental support [3.00 (3.00, 3.83) vs. 3.00 (2.83, 3.67), *p* < 0.01], autonomous motivation [3.50 (3.00, 4.50) vs. 3.50 (3.00, 4.00), *p* < 0.01], and the home environment (i.e., availability of PA resources) [1.88 (1.38, 2.50) vs. 1.50 (1.25, 2.00), *p* < 0.01].

**Table 2:** Descriptive statistics of moderate to vigorous physical activity, parental support, availability of physical activity resources in the home environment, and autonomous motivation by gender.

	Median (Interquartile Range)			*p*
	Total	Boy	Girl	
Meet the recommended criteria/ week (n, %)	3717, 99.44%	1980, 99.40%	1737, 99.48%	<0.001
MVPA (min/week)	801.21 (672.49, 936.63)	812.49 (686.94, 945.91)	787.34 (686.94, 945.91)	<0.001
Parental support	3.00 (2.83, 3.67)	3.00 (3.00, 3.83)	3.00 (2.83, 3.67)	<0.001
Autonomous motivation	3.50 (3.00, 4.00)	3.50 (3.00, 4.50)	3.50 (3.00, 4.00)	<0.001
Availability of physical activity resources in the home environment	1.63 (1.25, 2.25)	1.88 (1.38, 2.50)	1.50 (1.25, 2.00)	<0.001

Note: MVPA, moderate to vigorous physical activity. World Health Organization recommends that teenagers do at least 60 min of moderate to vigorous physical activity every day.

## Data Availability

Data are available from the corresponding author upon reasonable request. The data are not publicly available due to privacy restrictions.
